# Time-dependent effectiveness of the intracanal medicaments used for pulp revascularization on the dislocation resistance of MTA

**DOI:** 10.1186/s12903-015-0117-4

**Published:** 2015-10-23

**Authors:** Tugba Turk, Beyza Ozisik, Berdan Aydin

**Affiliations:** Department of Endodontology, Ege University, School of Dentistry, Izmir, Turkey; Department of Restorative Dentistry, College of Dentistry, University of Illinois at Chicago, 801 S. Paulina St, Room 531, Chicago, IL 60612 USA

**Keywords:** Regenerative endodontic treatment, Antibiotic pastes, MTA, Intracanal medicaments

## Abstract

**Background:**

The aim of the present study was to evaluate the time-dependent effectiveness of the intracanal medicaments used in pulp revascularization on the dislocation resistance of mineral trioxide aggregate (MTA).

**Methods:**

One hundred ninety-two extracted human maxillary incisor teeth were sectioned apically 12 mm below and coronally 2 mm above the cemento-enamel junction. Roots were enlarged to size 40 (Protaper F4). Next, Peeso reamers from #1 to #5 were used sequentially. Sodium hypochlorite (2.5 %), EDTA (17 %), and distilled water were used in final irrigation. The specimens were randomly divided into four groups (*n* = 48): Group 1, in which triple antibiotic paste (TAP) (ciprofloxacin + metronidazole + minocycline) was prepared and delivered into the canals using a lentulo spiral; Group 2, in which double antibiotic paste (DAP) (ciprofloxacin + metronidazole) was placed into the canals; Group 3, in which calcium hydroxide paste (CH) (calcium hydroxide + distilled water) was introduced into the roots; and Group 4 (control), in which no medicament was applied into the root canals. Then, the samples were kept in saline solution for 2, 4, and 12 weeks, after which time 16 roots were selected randomly from each group, representing the samples of each time point. After removal of the medicaments, MTA was placed into the coronal third of the roots, and the samples were incubated for 7 days. A push-out test was used to measure the dislocation resistance (DR) of MTA. The data were analyzed using a two-way ANOVA followed by Tukey’s pairwise comparisons (*p* = 0.05).

**Results:**

The time factor displayed a significant effect on the DR of MTA (*p* < 0.05). All medicaments resulted in significantly smaller DR values after 12 weeks compared to after 1 week (*p* < 0.05). A significant unfavorable effect of TAP and DAP was observed as early as 2 weeks after the application, while 2 and 4 weeks after the application of CH there was no effect on the DR of MTA. No significant differences were found between the time points in the control group (*p* > 0.05).

**Conclusion:**

The type and the intracanal duration of medicaments used for pulp revascularization should be chosen carefully to provide maximum antimicrobial effect while creating a favorable environment both for stem cell attachment and MTA adhesion.

## Background

Regenerative endodontic treatment (RET) is a tissue engineering concept specific to pulp and dentin regeneration inside the root canal space of devital teeth [1] in which the pulp tissue is completely removed due to a previous irreversible inflammatory response and microbial invasion. Successful results of RET of immature teeth including control of infection and root development have been shown in many case reports and case series [2–7]. To avoid further weakening of immature teeth, minimal mechanical instrumentation is recommended [8], but a sterile environment is necessary for pulp tissue regeneration in RET [9, 10]. Thus, disinfection of root canals is attempted only with irrigation solutions and intracanal medicaments [8, 11, 12]. Antibiotic pastes (metronidazole and ciprofloxacin with or without minocycline) and calcium hydroxide (CH) are commonly used medicaments for RET [6, 13–17].

While a 2- to 4-week treatment period is deemed sufficient to obtain the therapeutic effects of antibiotic pastes and CH [4–7, 14, 18–24], varying treatment periods from 1 week [3] to several months [5] were used in some case reports. However, alterations of the surface properties and deterioration of the mechanical properties of root canal dentin have been previously demonstrated when endodontic regeneration medicaments were used for long-term periods [25–28].

After the treatment period, MTA was generally placed on the coronal part of the root canal [4, 6, 18, 21, 22, 24]. MTA is a biocompatible, conductive, and inductive calcium silicate-based material that is able to bond to dentin chemically [29]. It is reported that pretreatment of dentin either with irrigants or medicaments may influence the bond strength of MTA [30, 31]. Bond strength is an important factor since teeth are exposed to occlusal and procedural forces that might dislodge the MTA after its placement [32–34]. Recently, Topcuoglu et al. [31] reported that the application of medicaments used in regenerative endodontics for 3 weeks affects the bond strength of MTA placed in root canal dentin. However, the effect of the intracanal duration of medicaments on the dislocation resistance (DR) of MTA has not yet been evaluated.

Therefore, the aim of the present study was to evaluate the time-dependent effectiveness of the intracanal medicaments used in pulp revascularization on the DR of MTA.

## Methods

The study protocol was approved by the Ethics Committee of Ege University (Protocol No. 15-1/9) and written informed consent form was obtained from each patient. One hundred ninety-two human maxillary incisor teeth with a single straight root canal, extracted in Department of Oral & Maxillofacial Surgery, were collected for this study. Reasons for extraction were periodontal diseases, such as insufficient periodontal support. The intact teeth were stored in thymol solution (0.1 %) for up to 3 months, observed carefully under 20x magnification, and excluded if resorption, caries, cracks, or fractures were detected.

Buccolingual and mesiodistal dimensions of teeth at the cemento-enamel junction were measured with a digital caliper (Mitutoyo, Tokyo, Japan) to provide standardization, and the mean mesiodistal and buccolingual dimensions were obtained. Thereafter, teeth deviating 20 % in their dimensions compared to the standard values were excluded.

### Sample preparation

Teeth were sectioned apically 12 mm below and coronally 2 mm above the cemento-enamel junction with a low-speed rotary saw (Isomet, Buhler, Lake Bluff, IL, USA). Tissue remnants were removed using a size 20 Hedström file (Dentsply Maillefer, Ballaigues, Switzerland)—samples were instrumented with rotary files (ProTaper, Dentsply, Maillefer, Ballaigues, Switzerland) to standardize the master apical file to be size 40 (F4). Next, Peeso reamers (Maillefer, Ballaigues, Switzerland) from #1 to #5 were used sequentially to obtain larger root canals.

Between the uses of each file, the root canals were irrigated with 2 mL of 2.5 % sodium hypochlorite (NaOCl) (Sigma-Aldrich, St. Louis, MO). Final irrigation was performed using 5 mL of 2.5 % NaOCl (1 min) and 5 mL of 17 % EDTA (Sigma) (1 min). Next, the canals were rinsed with sterile distilled water and then dried with paper points (Meta, Metabiomed, Chungbuk, Korea).

### Placing intracanal medicaments

The specimens were randomly divided into four groups: TAP, DAP, CH, and control, with 48 samples in each group. No medicament was used in the control group.

1. TAP paste: 1:1:1 mixture of ciprofloxacin (Bayer, Leverkusen, Germany), metronidazole (Sanofi Aventis, Frankfurt, Germany), and minocycline (Ratiopharm, Ulm, Germany) prepared with sterile distilled water (w/v 3:1), as described by Yassen et al. [35], was delivered into the canals using a lentulo (Dentsply Maillefer, Ballaigues, Switzerland).
2. DAP paste: 1:1 mixture of ciprofloxacin (Bayer) and metronidazole (Sanofi-Aventis) prepared with sterile distilled water (w/v 2.5:1) was delivered into the canals as described previously.
3. CH paste prepared by mixing calcium hydroxide powder (Ca(OH)_2_, Merck, Darmstadt, Germany) with sterile distilled water (w/v 2:1) was placed into the root canals as described previously.

The apical root canal orifices were sealed with modeling wax (Dentsply DeTrey, Bois Colombes, France), and the coronal orifices were sealed with glass ionomer cement (Fuji, GC, Tokyo, Japan), including the control group samples (*n* = 48). However, no medicament was applied in the root canals of the control group. All samples were kept in saline solution, which was replenished every 7 days to avoid dehydration throughout the evaluation period, which lasted up to 12 weeks. After 2, 4, and 12 weeks, 16 teeth were selected randomly from each group, representing the samples of each time point. At the end of the incubation period, the temporary filling material was removed with a size 3 round bur (Dentsply Maillefer) under water cooling, followed by the removal of TAP, DAP, and CH using 2 mL of 2.5 % NaOCl and 17 % EDTA. The root canals were then dried using absorbent paper points.

MTA (ProRoot, Dentsply Tulsa Dental, Tulsa, OK) was prepared in accordance with the manufacturer’s instructions and placed 4 mm deep into the coronal third of the roots with an amalgam carrier having a 4 mm-long chamber. Also, a saline-moistened cotton pellet was placed on top of the MTA to accelerate its homogenous setting. After the radiographic control of MTA placement, the samples were stored for a week at 37 °C at 100 % humidity to allow the setting of the MTA completely.

### Push-out test

After the completion of the storage periods of 2, 4, and 12 weeks, the coronal region of each root was horizontally sectioned to obtain a single slice from each root using an Isomet saw (Buehler, Lake Bluff, IL) under water cooling. Slice thickness was adjusted to 1 mm ± 0.1 mm utilizing a digital caliper (Mitoyo, Tokyo, Japan), while the root canal space was filled with cement material (MTA). Next, a 1.2 mm diameter test-tip was positioned over the cement so that it touched only the MTA and did not stress the root canal walls.

The force was applied at a crosshead speed of 1 mm/min using a universal testing machine (AGS-X, Schimadzu Co, Kyoto, Japan) until failure occurred. The force (N) required to dislocate the MTA was recorded in Newtons. To express the push-out bond strength in MPa, the load at failure (N) was divided by the area of the adhesion surface, calculated by the following formula: 2 πr × h, where π is the constant 3.14, r is the root canal radius, and h is the thickness of the root slice in millimeters.

### Statistical analysis

The effects of the type of medicament used and the duration of the medicament treatment on the DR of MTA were examined using a two-way ANOVA followed by Tukey’s pairwise comparisons. A 95 % confidence interval was applied to determine statistical significance.

## Results

Mean DR values (MPa) after the push-out tests are shown in Table 1. No significant difference was found between the control group at all time-points (*p* > 0.05). The time factor displayed a significant effect on the DR of some experimental groups; prolonged treatment time caused a decrease in DR values. All medicaments resulted in significantly smaller DR values after 12 weeks compared to a 2-week treatment time (*p* < 0.05). The 12-week treated samples in the TAP, DAP, and CH groups had significantly smaller DR values compared to the control group (*p* < 0.05). The 2- and 4-week-treated samples in the TAP and DAP groups had significantly smaller DR values compared to the control group. There were no statistically significant differences between the control and CH treated groups with 2- and 4-week treatment periods (*p* > 0.05). Within the experimental groups, the DAP group had the smallest DR values while the CH group had the highest.

**Table 1:** Mean DR values of groups

Group	Mean ± SD (MPa)		
	2-week (*n* = 18)	4-week (*n* = 18)	12-week (*n* = 18)
TAP	5.19 ± 0.79^a,c^	4.47 ± 0.67^a,b,d^	2.98 ± 0.84^a^
DAP	4.90 ± 0.69^a,b,d^	4.21 ± 0.76^a,b,d^	2.94 ± 1.01^a^
CH	5.64 ± 0.59^d^	5.59 ± 0.66^d^	3.53 ± 0.99^a^
Control	5.70 ± 0.68	5.6 ± 0.51	5.54 ± 0.68

^a^Significant difference with control group

^b^Significant difference with CH group

^c^Significant difference with 4 and 12-week

^d^Significant difference with 12-week

## Discussion

Since its introduction, MTA has been regarded as an ideal material for many dental applications such as pulp capping, repair of perforations, apexification, and lately in RET [29, 32]. In addition to its biological impacts on tissue healing and regeneration, the DR of MTA should be satisfactory [30, 32, 34, 36] since the DR of such materials is one of the factors that affect sealing ability [30].

MTA is subjected to forces that arise during restorative procedures or mastication when it is placed into the coronal part of the root canal [31, 37]. The push-out test has been shown to be a reliable method for evaluating the DR of materials [38], and it is utilized in many studies [31, 33, 37, 39]. The dentin bonding strength of MTA has been previously evaluated while considering modifications in organic and inorganic dentin compositions that affect the DR of MTA. DR might be influenced by MTA thickness, humidity of the surrounding tissues, evaluation time, alkaline pH of the environment, and physical properties of the contacting dentin surface [30, 31, 37, 39]. Evaluation of the DR of MTA has become crucial as the dentin surface of the root canal is exposed to various irrigation solutions and intracanal medicaments, which may alter its chemical and mechanical properties during pulp revascularization procedures or conventional endodontic treatment. Recently, the medicaments used in pulp revascularization were evaluated in regard to their influence on the DR of MTA placed into the root canal dentin. It was found that the application of DAP reduced the DR of MTA, whereas TAP and CH did not affect the DR after a 3-week period [31]. However, according to the results of the present study, it is noteworthy to mention that DR was significantly affected when TAP intracanal medicament was kept longer than 2 weeks and the CH intracanal medicament was kept in the root canals longer than 4 weeks. Effects of medicaments on the DR of MTA were compared based on the various treatment periods in the previous case reports, which lack consensus in terms of the optimal treatment period for pulp revascularization. According to the results of the present study, the DR of MTA was influenced by the use of different type of medicaments and various treatment time periods. Antibiotic pastes seemed to affect the DR of MTA in an undesirable manner. The use of DAP decreased the DR in 2 weeks and also caused a further gradual decrease of DR. On the other hand, DR was gradually and significantly decreased when TAP was used in the root canals for 4 to 12 weeks. The effect of TAP and DAP on dentin was evaluated by Yassen et al. [27], who showed that antibiotic pastes have a deteriorating effect that increases in a time-dependent manner due to their acidic nature, which can demineralize dentin. Nonetheless, it was also shown that the application of CH may cause the collagen degradation of dentin, justifying reduction in the DR of MTA [28]. Recently, significant increases in the surface roughness and alterations in the inorganic phase of dentine like Ca and P were demonstrated with the use of TAP, DAP, and CH on dentine [40, 41].

Demineralization, erosion, and surface roughness of dentin may decrease the initial mechanical adhesion established between MTA and dentin; however, adhesion transforms into a chemical binding by a diffusion-controlled reaction between the apatite layer of MTA and dentin [29]. The physicochemical interaction of MTA with endodontically prepared root canal dentin was studied by Sarkar et al. [29], who found that MTA leaches some ions, of which Ca^2+^ is the most abundant. This leads to the formation of an interfacial layer (hydroxyapatite or carbonated apatite), and further examination of this adhesion layer revealed a structure similar in composition to hydroxyapatite that is composed of calcium, phosphorus, and oxygen [29]. The formation of an interfacial layer on MTA is an important factor for such biomaterials because, when MTA comes into close contact with calcified tissues, it forms a chemical bond by means of this interfacial layer [42–44]. It has been speculated that triggering the formation of an interfacial layer between MTA and dentin with medicaments might create a favorable sealing ability as the adhesion and dislodgement resistance of MTA would be enhanced [36, 45, 46]. When phosphate-buffered saline is used as an intracanal dressing after MTA placement, enhanced biomineralization of MTA is observed as the formation of an interfacial layer with tag-like structures [36].

In the present study, adhesion of MTA on CH-treated dentin was greater than the adhesion of MTA on antibiotic-treated dentin at 2 and 4 weeks. Shokounejad et al. [33] suggested the application of non-setting CH to neutralize the pH and to increase the DR of MTA prior to the placement of MTA in a low pH environment. One-week CH treatment was shown to improve the marginal adaptation of the MTA apical plug [47]. This improvement was attributed to the conversion of calcium hydroxide to calcium carbonate or the reaction of MTA with residual calcium hydroxide. Thus, in the current study, the favorable results in the CH-treated group after 2 to 4 weeks may be related to the Ca^2+^ ions supplied by the residual CH, a phenomenon that has been previously mentioned to explain the improved adhesion of MTA.

A study compared the performance of CH and TAP removal from root canals, demonstrating that the residues of CH were distributed rather superficially and contained a smaller amount of material [48]. On the other hand, TAP appeared to have greater retention and deeper penetration; thus, it was more difficult to remove. However, while residual TAP may lead to demineralization of dentin via decreasing the pH, it may also chelate the calcium in dentin, which is reported to be significant in establishing the adhesion of MTA on dentin [27, 33, 49].

CH caused a significant decrease of the DR of MTA after 12 weeks of treatment. However, 2- and 4-week applications did not exhibit such an effect, indicating the safety of short-term CH use. The undesired effects of long-term CH application on the mechanical properties of dentin have been reported by several researchers [28, 50, 51]. The effect of TAP, DAP, and CH on fracture resistance and microhardness of root canal dentin was investigated, and the longevity of the treatment time was found to have a significant jeopardizing effect on both microhardness and the fracture resistance of roots [28]. Moreover, all type of medicaments significantly decreased the fracture resistance of roots after a 3-month period compared to 1-week application. The application of antibiotic paste significantly decreased the microhardness of dentin after 1- or 3-month periods, whereas 1-week application did not. CH increased the microhardness of dentin in all treatment periods. Therefore, short-term use of the root canal medicaments is suggested to prevent further weakening of the immature root structures. However, root cylinders were left empty after placing the medicaments, and MTA was not used in latter study. Thus, no interpretation can be drawn from their results regarding the DR of MTA.

## Conclusions

All medicaments decreased the DR of MTA after a 12-week application period. However, a significant decreasing effect of TAP and DAP was observed as early as 4 weeks, while 2- and 4-week application of CH did not effect the DR of MTA. The type and the treatment time of medicaments kept in root canals used for pulp revascularization should be optimized to provide maximum antimicrobial effect while creating a favorable environment both for stem cell attachment and MTA adhesion.

## Abbreviations

MTA: Mineral trioxide aggregate
DR: Dislocation resistance
TAP: Triple antibiotic paste
DAP: Double antibiotic paste
CH: Calcium hydroxide
EDTA: Ethylene diamine tetraacetic acid
NaOCl: Sodium hypochlorite
mL: Mililiter
MPa: Megapascal
